# Transgenic Quail Production by Microinjection of Lentiviral Vector into the Early Embryo Blood Vessels

**DOI:** 10.1371/journal.pone.0050817

**Published:** 2012-12-12

**Authors:** Zifu Zhang, Peng Sun, Fuxian Yu, Li Yan, Fang Yuan, Wenxin Zhang, Tao Wang, Zhiyi Wan, Qiang Shao, Zandong Li

**Affiliations:** 1 State Key Laboratories for Agrobiotechnology, China Agricultural University, Beijing, China; 2 Beijing Education Examinations Authority, Beijing, China; University of Connecticut, United States of America

## Abstract

Several strategies have been used to generate transgenic birds. The most successful method so far has been the injection of lentiviral vectors into the subgerminal cavity of a newly laid egg. We report here a new, easy and effective way to produce transgenic quails through direct injection of a lentiviral vector, containing an enhanced-green fluorescent protein (eGFP) transgene, into the blood vessels of quail embryos at Hamburger-Hamilton stage 13–15 (HH13–15). A total of 80 embryos were injected and 48 G0 chimeras (60%) were hatched. Most injected embryo organs and tissues of hatched quails were positive for eGFP. In five out of 21 mature G0 male quails, the semen was eGFP-positive, as detected by polymerase chain reaction (PCR), indicating transgenic germ line chimeras. Testcross and genetic analyses revealed that the G0 quail produced transgenic G1 offspring; of 46 G1 hatchlings, 6 were transgenic (6/46, 13.0%). We also compared this new method with the conventional transgenesis using stage X subgerminal cavity injection. Total 240 quail embryos were injected by subgerminal cavity injection, of which 34 (14.1%) were hatched, significantly lower than the new method. From these hatched quails semen samples were collected from 19 sexually matured males and tested for the transgene by PCR. The transgene was present in three G0 male quails and only 4/236 G1 offspring (1.7%) were transgenic. In conclusion, we developed a novel bird transgenic method by injection of lentiviral vector into embryonic blood vessel at HH 13–15 stage, which result in significant higher transgenic efficiency than the conventional subgerminal cavity injection.

## Introduction

The study of birds makes important contributions to scientific knowledge. Birds are used as animal models in biological research and the avian oviduct can serve as a bioreactor for the production of valuable proteins in the egg [1]. While avian transgenic technology has advanced markedly in recent decades, the production of transgenic birds is still not as efficient as the production of transgenic mammals due to the characteristics of bird development [2]. A chicken egg contains 40,000–80,000 undifferentiated blastoderm cells with a large amount of yolk [3], making transgenic manipulations difficult. In the past ten years, different strategies have been used in the production of transgenic birds, including zygote, blastoderm, primordial germ cell (PGC), and testicular cell-based methods [2], [4]. Traditionally, researchers preferred to produce transgenic birds by injection of lentiviral vectors into the subgerminal cavity of newly laid eggs at stage X of avian embryo developmental stage (Figure S1) [5], [6], [7], [8], [9], [10]. Recently, Lyall *et al.* have successfully generated transgenic chickens that were resistant to avian influenza [11]. The infection of these blastoderm cells in subgerminal cavity by lentivirus successfully produced G0 transgenic chimeras and transgenic G1 birds, but random integration of the transgene frequently resulted in transcriptional blockade [4].

PGCs are the precursors of sperms and eggs which have been suggested to be the most appropriate tool for cell-mediated gene transfer in comparison with the conventional blastoderm cell-based methods [4], [12]. The migration of PGCs in the chicken embryo has been clearly defined. PGCs first arise from the epiblast and migrate to the hypoblast of the pellucida (germinal crescent) at Hamburger–Hamilton stage 4 (HH4, HH stages start after egg laid at the end of Stage X in avian embryo development). Between HH10–12, PGCs move from the germinal crescent into the bloodstream [13], [14] and migrate through the circulatory system until they finally settle in the genital ridges [15], [16]. The characteristic migration of PGCs through the bloodstream at HH10–17 facilitates the production of germ line chimeras by using circulating PGCs as targets for transgene integration [4]. Germ line chimeras were previously generated by injecting PGCs, collected from the bloodstream of embryos at HH13–17, into recipient embryos [17], [18]. Van de Lavoir *et al.* successfully produced transgenic chickens using PGCs derived from embryonic blood. Shin *et al.* produced transgenic quails via a germline transmission system using post-migratory gonadal primordial germ cells (gPGCs) from the embryonic gonads of 5 day-old birds [10], [12].

As an improvement to the previous approaches, we have developed a novel method incorporating the injection of a lentiviral vector directly into the blood vessels of HH13–15 quail embryos which avoids the manipulation of PGCs *in vitro*. This method generates transgenic germ line chimeras, that are able to produce transgenic offsprings. To compare our new method with the established approach, the same volume of lentivirus was microinjected into the subgerminal cavity of stage X blastodermal embryos. Our new method achieved a higher efficiency of transgenesis than the conventional method. This new approach should make it easier to manipulate the avian genome and may lead to novel biotechnological applications.

## Materials and Methods

### Lentiviral vector

**Figure 1:**
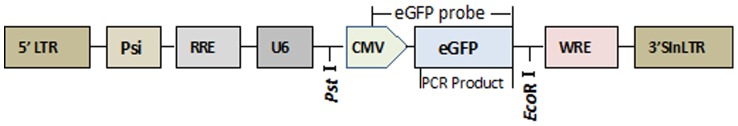
Diagram of the relevant regions of the pGCL-eGFP vector used to generate chimeric quail. LTR, long terminal repeat; Psi, packaging signal; RRE, rev responsive element;U6,U6 promoter; CMV, cytomegalovirus promoter; eGFP, enhanced green fluorescent protein gene; WRE, woodchuck hepatitis virus post-transcriptional regulatory element; SinLTR, self-inactivating LTR. The Southern blot probe and PCR product to detect the eGFP gene are indicated. *Pst*I and *Eco*RI restriction enzymes were used for Southern blot analysis.

The lentiviral suspension was purchased from GeneChem Biotechnology Co. Ltd (Shanghai, China). The titer of the lentiviral stock was 2×10^9^ titer units (TU)/ml. A schematic representation of pGCL-eGFP within the lentiviral vector is shown in Figure 1. The sequences of the Lentiviral vector used in this study are shown in Text S1.

### Egg incubation

Freshly laid Japanese quail eggs were purchased from Deling Quail Farm (Beijing, China). The upper surface of the eggs was washed with 0.1% benzalkonium bromide and sprayed with 70% ethanol for sterilization. Eggs were incubated blunt end up at 37.5°C, in a relative humidity of 55–65%, while being rocked at a 90° angle at 2 h intervals.

### Microinjection of the lentiviral vector and animal care

The lentiviral stock was first diluted 1∶1 with DMEM. In blood vessel injection groups, freshly laid eggs were incubated for 46–48 h to make the embryos develop to HH13–15. The embryo was then located under a fiber optic light source and its location was marked. A window (3×3 mm^2^) in the shell was opened using a dental drill and tweezers. One microliter of viral suspension at 1×10^9^ TU/ml, diluted from stock solution (2×10^9^ TU/ml), was injected into the blood vessels of HH13–15 recipients using a glass micropipette (tip diameter: 40–50 µm) under a stereo microscope (Figure S2). The window was sealed with Parafilm®, and the injected eggs were incubated until hatching.

In subgerminal cavity injection groups, freshly laid, sterilized eggs were positioned horizontally for 4–6 h. A window (3×3 mm^2^) in the shell was opened using a dental drill and tweezers. One microliter (1×10^9^ TU/ml) of lentiviral vector was injected into the subgerminal cavity of the blastoderm of stage X blastodermal embryos (Figure S3). The window was then sealed with Parafilm® and the eggs incubated until hatching. Non-injected eggs, hatched embryos and birds were used as negative controls in the present study.

Animal welfare and experimental procedures conformed to the Institutional Guidelines of the Care and Use of Laboratory Animals at China Agricultural University (Beijing, China). All efforts were made to minimize animal suffering and the number of animals used.

### Polymerase chain reaction (PCR) analysis

To identify transgenic quail, genomic DNA was extracted from G1 blood and G0 semen using a genomic DNA purification kit (Promega, San Luis Obispo, CA, USA). PCR analysis was carried out in a volume of 25 µL containing 12.5 µL 2× Perfect Shot™ Ex Taq PCR premix buffer (TaKaRa, Tokyo, Japan), 50 ng genomic DNA, and 10 pM of each primer. Primers used to amplify the eGFP sequence of the lentiviral vector, pGCL-eGFP, were 5′-CGGCCACAAGTTCAGCGTGTC-3′ and 5′-CGATGGGGGTGTTCTGCTGGT-3′. After initial denaturation at 94°C for 5 min, 35 cycles of amplification were performed. DNA was denatured at 94°C for 30 s, annealed at 58°C for 30 s, and extended at 72°C for 45 s. The samples were incubated at 72°C for 10 min to ensure complete strand extension. pGCL-eGFP plasmid DNA was used as a positive control.

### Southern blotting

Ten to twenty micrograms of genomic DNA was extracted from positive and negative G1 offspring as described for PCR. Genomic DNA was digested with *Eco*RI and *Pst*I overnight at 37°C. DNA fragments were fractionated by 1% agarose gel electrophoresis and transferred to nylon membrane. The transgene was detected using a 876 bp DIG-labeled probe (DIG High Prime Lab/Det Kit II; Roche Diagnostics, Mannheim, Germany) against the eGFP sequence (Figure 1).

### Fluorescence imaging

eGFP expression in embryos and mature quail tissues was visualized using an IX71 fluorescence microscope (Olympus, Tokyo, Japan). Digital images were captured using a DP70 camera (Olympus).

### Statistical analysis

Statistical comparison was performed using Mann-Whitney U-test. A *P* value less than 0.05 was considered statistically significant. The p values in Table 1 were derived from the comparison between vessel injection vs subgerminal cavity injection group regarding hatching rate, gender ratio in G0 generation, the semen PCR positive rate of G0 and transgenic efficiency of G1 generation.

**Table 1:** Comparison of subgerminal cavity injection and blood vessel injection for the production of transgenic quails.

Injection methods	Hatchability	Sex ratio of G0 quails	Semen PCR positive ratio of G0	Transgenic efficiency of G1
**Subgerminal cavity injection**	14.1% (34/240)	55.9% (19/34, male/total)	15.7% (3/19)	1.7% (4/236)
**Blood vesse linjection**	60.0% (48/80)	43.8% (21/48, male/total)	23.8% (5/21)	13.0% (6/46)
**P value**	<0.001	0.279	0.690	0.017

The p values were derived from the comparison between vessel injection method vs subgerminal cavity injection method.

## Results

### Transgene detection in chimeric quail

**Figure 2:**
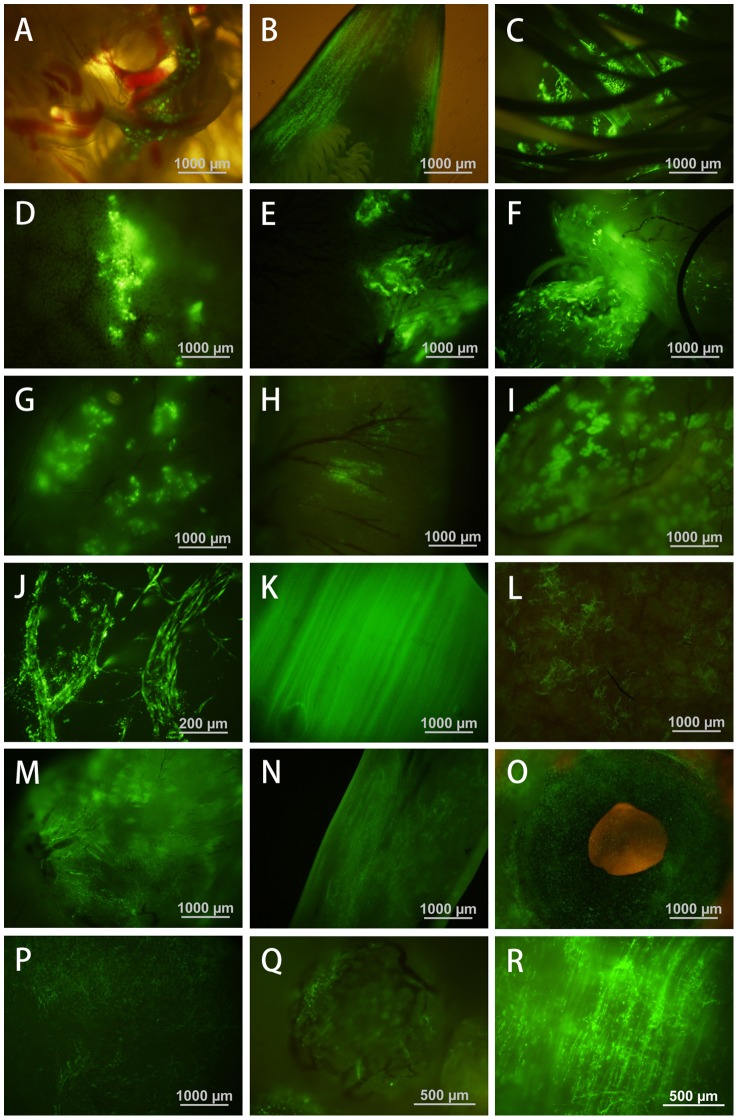
Expression of eGFP in tissues of hatched transgenic chimeric quail by fluorescence microscopy. Lentiviral vector was injected into the blood vessels of HH13–15 embryos. eGFP expression in (A) the vitelline membrane of 4 day-old embryo; and in (B) the beak, (C) feather, (D) liver, (E) kidney, (F) mesenterium, (G) glandular stomach, (H) small intestine, (I) large intestine, (J) blood vessel, (K) breast muscle, (L) spleen, (M) heart, (N) oviduct, (O) eye, and (P) brain of newly hatched G0 quails; and in (Q) the follicle of sexually matured G0 quail, (R) the skeletal muscle of G1 progeny.

Quail embryos at stage HH13–15 were chosen to injecte the lentiviral vector containing the eGFP gene driven by a cytomegalovirus (CMV) promoter, because the blood vessels are too fine to inject with the microinjection pipette before HH 13–15 while the transgene expression in the gonads was greatly reduced or completely absent after these stages. Eighty embryos were injected and 48 quails were successfully hatched (60.0% hatchability, Table 1). eGFP expression can be detected in many different tissues in all embryos and hatched quails in pilot experiments. In Figure 2A eGFP expression was shown as the presence of green fluorescence in the vitelline membrane of 4 day-old embryos. In newly hatched quails, expression was detected in the beak, feather, liver, kidney, mesenterium, glandular stomach, small intestine, large intestine, blood vessels, breast muscle, spleen, heart, oviduct, eye and brain (Figure 2 B–N, P, Q).In sexually matured quails eGFP was expressed in the follicles (Figure 2O) and strong expression was also detected in the breast muscle and heart (Figure 2K, M).

In another experiment, we injected the same lentiviral vector into the subgerminal cavity of 240 quail embryos at stage X, total 34 were hatched that were much lower than blood vessel injection (14.1% hatchability, p<0.01). Similar to blood vessel injection, ubiquitous eGFP expression by subgerminal cavity injection can also be detected in in day 3 and day 6 embryos as well as in newly hatched birds (data not shown). Both injection methods produced viable male and female G0 birds at sex ratio of 55.9% and 43.8% for subgerminal and blood vessel injection, respectively. To generate G1 quails, 3 and 5 males with positive eGFP in semen from each injection group were mated with non-transgenic female birds.

### Transgene detection in the gonads of chimeric quail

**Figure 3:**
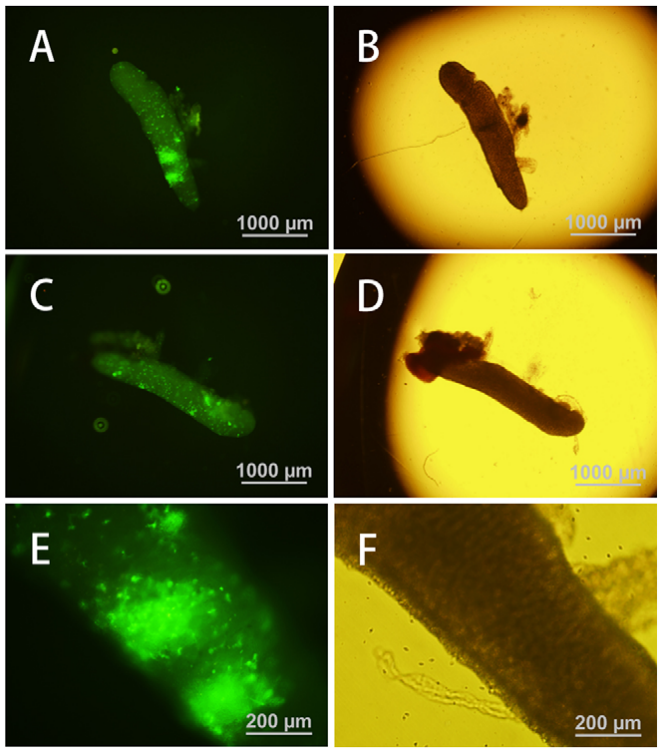
Expression of eGFP in the gonads. Lentiviral vector was injected into the blood vessels of HH13–15 embryos. Newly hatched quails were observed by fluorescence microscopy. (A, B, C, D) Gonads of a 3 day-old male quail left and right. (E, F) Enlargement of part of the gonads. (B, D, F) are bright-field images.

**Figure 4:**
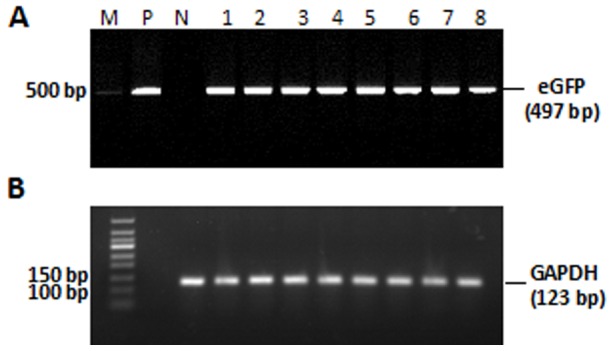
PCR analysis of genomic DNA extracted from semen of transgenic germ line chimeric (G0) quail. (A) PCR product of eGFP; (B) PCR product of housekeeping gene GAPDH; (1–5) PCR products of sexually matured G0 males produced by microinjection to blood vessels of embryo at HH Stage 13–15 (6–8). PCR analysis of three sexually mature males produced by microinjection beneath the subgerminal cavity of the blastodermal embryo. (M) Standard DNA markers. (P) Positive control. (N) negative control without microinjection of lentiviral vector. PCR product size is indicated on the right.

The blood vessel injection of the lentiviral vector at embryonic stage HH13–15 resulted in eGFP expression in the gonads of newly hatched and matured quails (Figure 3). The semen from five of 21 hatched male quail was positive for the eGFP gene, as determined by PCR (semen positive rate: 23.8%; Figure 4). This indicates that these five male quail were transgenic germ line chimeras.

**Figure 5:**
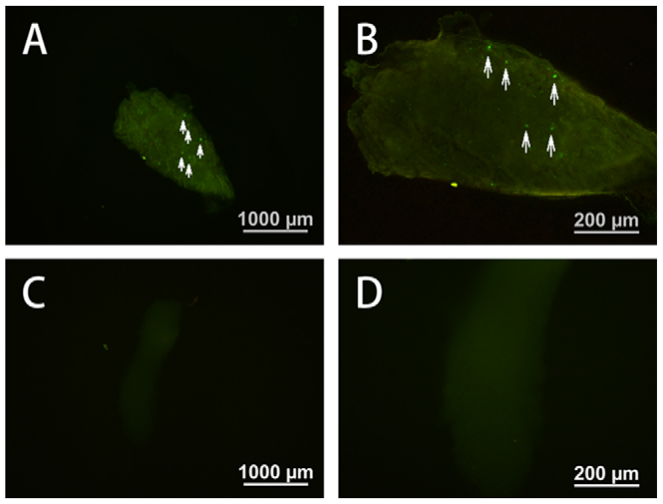
Expression of eGFP in the ovary. Lentiviral vector was injected beneath the subgerminal cavity of blastodermal embryos. Newly hatched quails were observed by fluorescence microscopy. (A, C) Ovary of a 3 day-old female quail. (B, D) Enlargement of part of the ovary. (C, D) are non-transgenic control.

Lentiviral vector was also injected into the subgerminal cavity of embryos at stage X to produce transgenic birds. In the gonads of both newly hatched and mature quails, eGFP expression was undetectable, but a few eGFP signals were detected in the ovary of new hatched quails (Figure 5). Semen was collected from 19 males, and only three samples were PCR-positive for eGFP (semen positive rate: 15.7%; Figure 4), indicating that these three males are transgenic germ line chimeras.

### Germline transmission of transgene to G1 quails

**Figure 6:**
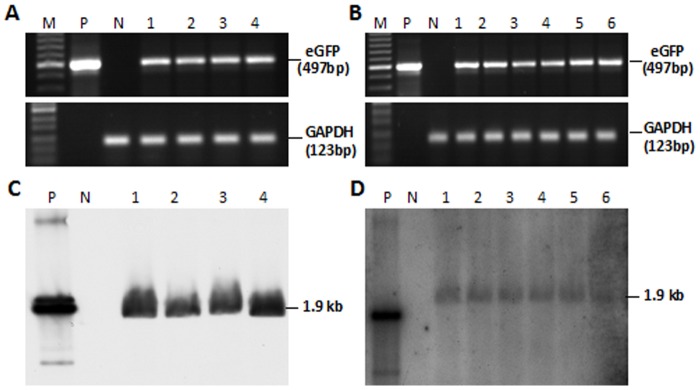
PCR and Southern blot analysis of genomic DNA from the blood of G1 transgenic quails. (A) PCR analysis of G1 offspring obtained by microinjection to the subgerminal cavity of blastodermal embryos. Lanes 1–4 represent the transgenic quails. (B) PCR analysis of five G1 offspring produced by microinjection into the blood vessels of HH13–15 embryos. Lanes 1–6 represent transgenic quails 1–6; MW, DNA size markers; P, positive control; N, negative control; PCR product size is indicated on the right. (C) Southern blot results. N, nontransgenic quail; Lanes 1–4 indicate G1 transgenic quails 1–4 (as in A). (D) Genomic DNA (10 µg) was digested with *Pst*I and *Eco*RI and hybridized with the eGFP probe. P, 80 pg of pGCL-eGFP vector; N, negative control without injection of lentiviral vector; Lanes 1–6 indicate G1 transgenic quails 1–6 (as in B).

The germline chimeras (G0) were mated with wild-type quails and to generate transgenic offsprings. The G1 progenies were screened by PCR and Southern blot analysis. In the offsprings of birds generated by blood vessel injection at embryonic stage HH13–15, 6/46 (13.0%) G1quails were eGFP positive (Figure 6B, D). Strong green fluorescence could also be observed in the skeletal muscle in one of the G1 birds died at day 23 after hatch (Figure 2R). In the offsprings of the birds by subgerminal cavity injection at stage X, only 4 out of 236 quails were eGFP positive (1.7%), the integration site of of eGFP gene in genome of the quails shows single by the Southern blot analysis (Figure S4). This was significantly lower than blood vessel injection (Table 1 and Figure 6A, C). Among 4 eGFP positive quails in 236 G1 chicks from subgerminal cavity injection there were 2 males and 2 females, while in 6 eGFP positive quails in 46 G1 chicks from blood vessel injection there were 4 males and 2 females.

## Discussion

Avian PGCs are progenitor cells of ova and spermatozoa. They have a characteristic heterochromatin nuclear marker, and are found in the epiblast of the avian embryo at stage X [19], [20], [21]. Following the formation of blood vessels at stage X, PGCs begin to migrate from the epiblast towards the hypoblast, and start to circulate through the bloodstream, which continues until HH stage 16. By HH stages 20–24 the PGCs have migrated into the gonadal anlage where they rapidly proliferate and differentiate into either spermatogonia in the testis or oogonia in the ovary [22], [23]. Aige-Gil and Simkiss showed that the number of PGCs in the bloodstream was maximal at HH stages 13–14 [24]. Based on this observation, we injected lentiviral vector carrying the eGFP gene directly into the yolk sac vessels of HH13–15 embryos to generate transgenic germ line chimeras. Kawabe *et al.* have successfully generated transgenic chickens through injection of a retroviral vector into the hearts of developing chicken embryos [25]. However, use of retroviral vectors often resulted in progeny with genetic mosaicism and viral silencing [26]. In addition, the injection of the virus into the heart requires expensive equipment and might cause vital damage to the embryos. In the present study, we modified this method by injecting the virus directly into the blood vessels. This approach reduces the risk of damage to the embryo. In addition, in this new method, we only opened a small window (3×3 mm^2^) near the blunt end of the egg. This can increase hatchability to about 60% (48/80) compared to the blastoderm-injected group, in which only 14.1% of quails hatched. This hatchability is higher than that of previous reports where injection of viral vector into the subgerminal cavity achieved a hatchability of 10–40% [5], [27], [28], [29]. This might be due to the embryo manipulation at different developmental stages and whether surrogate shells were used, as established by Perry [30].

In comparison of the transgenic efficiency between our approach and the conventional method, it was shown that G0 semen was transgene positive at the rate of 15.7% (3/19) for subgerminal cavity injection and 23.8% (5/21) for blood vessel injection (Table 1). Although the positive rate of the two methods was not significantly different, however, the rate of germline transmission of transgenes from G0 to G1 was low in subgerminal cavity injection, varing from 0.6%–1.6% by different investigators [8], [10]. We achieved similar rate (1.7%) using the same method. With blood vessel injection we achieved 13.0% transgene efficiency, nearly 8 times higher than the conventional method. We speculate that the maximal levels of PGCs in the blood at HH stage 13–15 may be the major contributing factor that would enhance the exposure of PGCs to lentiviral vectors, greatly increasing the infection rate of PGCs. Whereas in stage X, only small numbers of PGCs are present in the blood and they are also surrounded by blastoderm cells. Therefore, there is a reduced chance of exposure to the lentiviral vectors.

**Figure 7:**
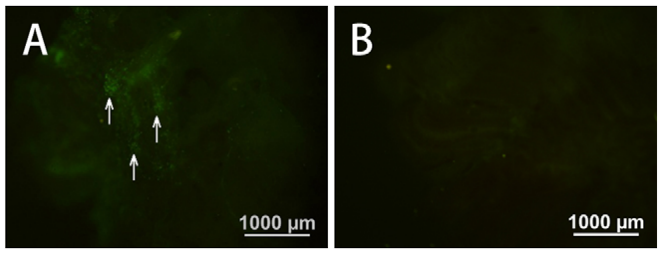
Fluorescent images of the lungs from quails. (A) the lung from a G0 quail from blood vessel injection group; (B) the lung of a non-injection control.

The lentiviral vector used in this study included eGFP, a CMV promoter, and a WRE (woodchuck hepatitis virus post-transcriptional regulatory element). The WRE is a post-transcriptional regulatory element that increases the efficiency of eGFP expression [31], [32]. Most tissues from transgenic chimeras expressed eGFP (Figure 2). The highest level of eGFP expression was found in the breast muscle and heart, and relatively lower in the lungs (Figure 7). McGrew *et al.* found that the eGFP gene, driven by a CMV promoter, was strongly expressed in the pancreas and weakly expressed in skeletal muscle and the heart [5]. These discrepancies could be due to the different methods of lentiviral vectors introduced into the embryo, or the influence of the WRE. The other issue to be addressed is that highly expressed transgene through PGCs infection via lentiviral vector may interfere with the normal development and physiological functions in the birds. For example, we found that high level expression of eGFP driven by a ubiquitin promoter resulted in the death of all G1 birds within 7 days after hatch (data not shown). However, we also noted that Kamikira et al generated transgenic chicken expressing human single chain Fv-Fc antibody at very high levels without any abnormality in the development and the physiological funtions in these birds [33].

In summary, we successfully produced germline transgenic quails by direct injection of lentiviral vector into the blood vessels of HH13–15 quail embryos. In comparison with conventional method of subgerminal cavity injection, our new method dramatically increased the germline transmission rate, presumably due to the direct contact of the lentiviral vector with PGCs circulating in the bloodstream. Furthermore, the new method was improved by avoiding the use of surrogate shells and the manipulation of PGCs *in vitro*. Therefore, infection of PGCs with lentiviral vector via direct injection into blood vessels has the potential to provide a more convenient and efficient way to produce transgenic birds.

## Supporting Information

Figure S1 **Structure of the freshly laid quail egg (Stage X) hatched with half shell removed.**
(TIF)

Figure S2 **A photo showing microinjection to blood vessel of a quail egg hatched for 46–48 hours at HH Stage 13–15.** The arrow indicates the embryonic abdominal aorta.
(TIF)

Figure S3 **A photo showing the microinjection to subgerminal cavity of a freshly laid quail egg at Stage X.** The arrow indicates the subgerminal cavity.
(TIF)

Figure S4 **Southern blot of the 4 positive G1 transgenic quails from Subgerminal cavity injection group.** The genomice DNA extracted from blood was digested with *Eco*RI. Probe used in this experiment was a 876 bp DIG-labeled probe asdescribe in materials and methods. N, nontransgenic quail; Lanes 1–4 indicate G1 transgenic quails 1–4.
(TIF)

Text S1 **The exact sequences of lentiviral vector pGCL-eGFP.**
(DOC)
