# Supplementary figures and images for: Transgenic Quail Production by Microinjection of Lentiviral Vector into the Early Embryo Blood Vessels
Source: PLoS One. 2012 Dec 12;7(12):e50817. doi: 10.1371/journal.pone.0050817 (PMC3520935; doi:10.1371/journal.pone.0050817)

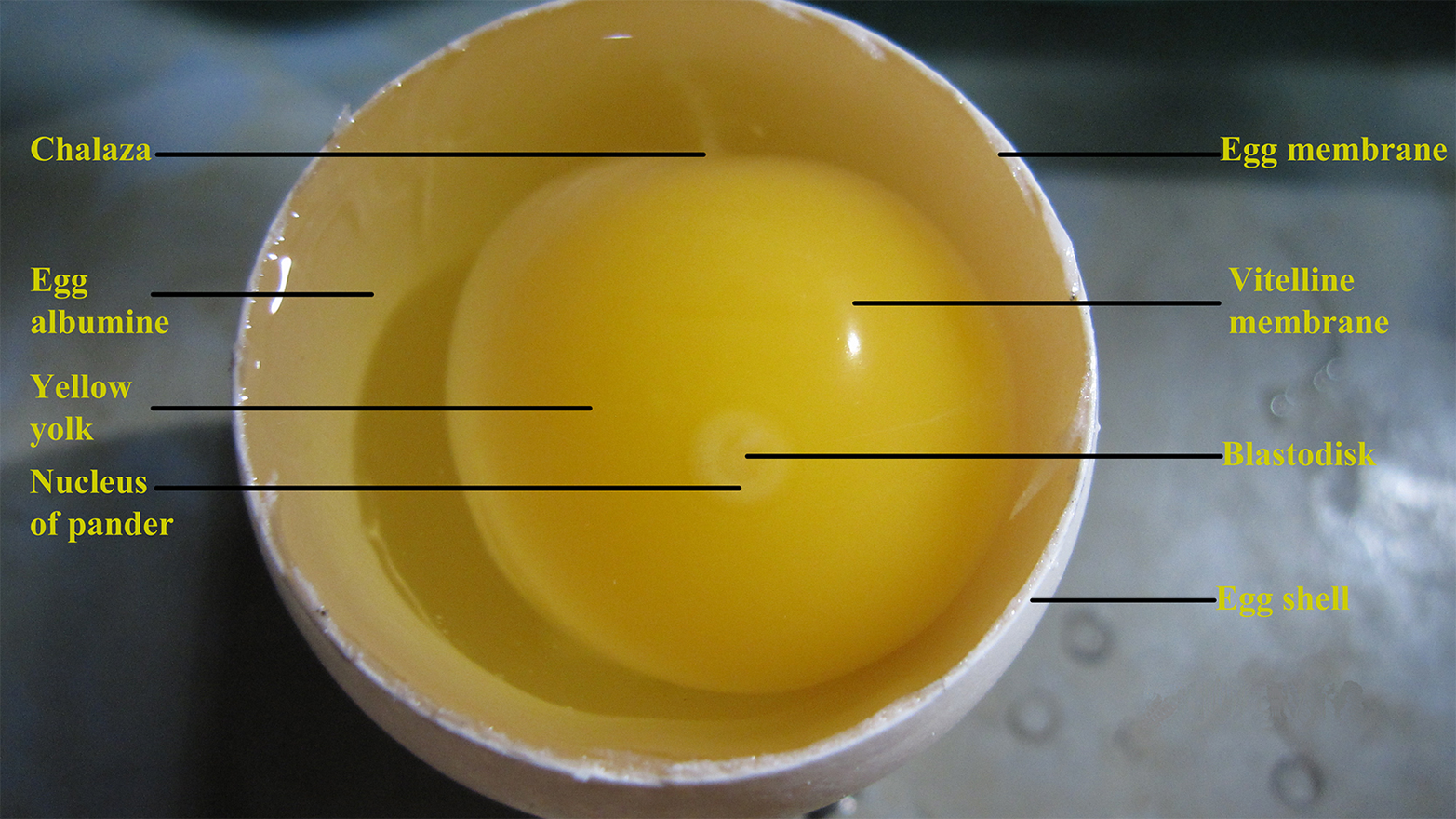

Supplement: Figure S1 — Structure of the freshly laid quail egg (Stage X) hatched with half shell removed. (TIF) [file pone.0050817.s001.tif]

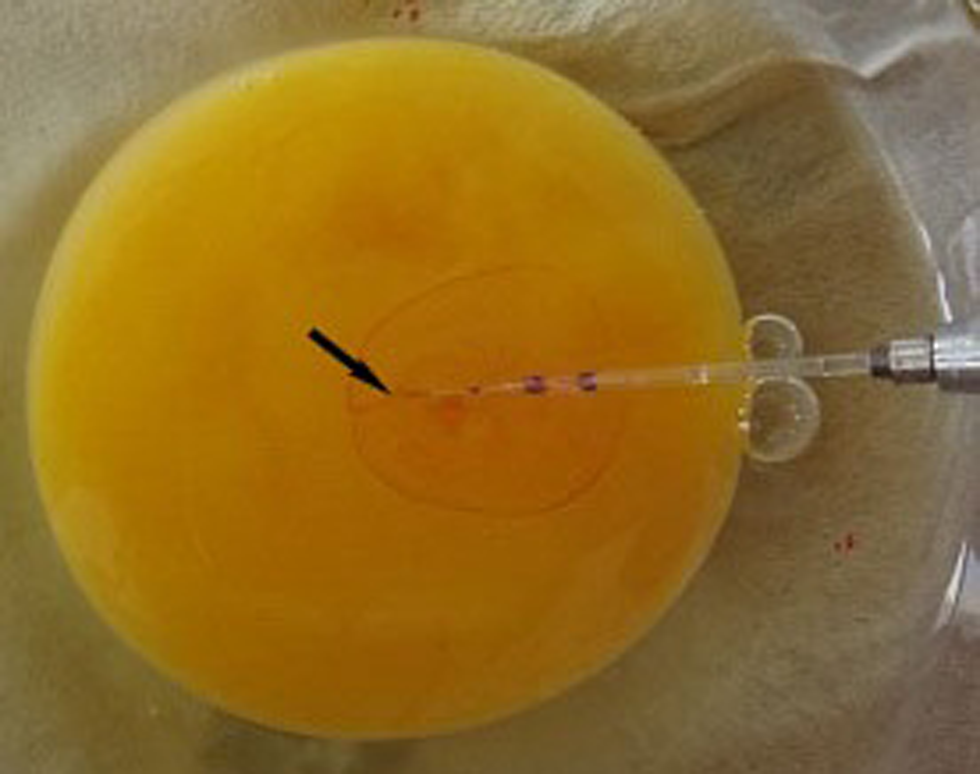

Supplement: Figure S2 — A photo showing microinjection to blood vessel of a quail egg hatched for 46–48 hours at HH Stage 13–15. The arrow indicates the embryonic abdominal aorta. (TIF) [file pone.0050817.s002.tif]

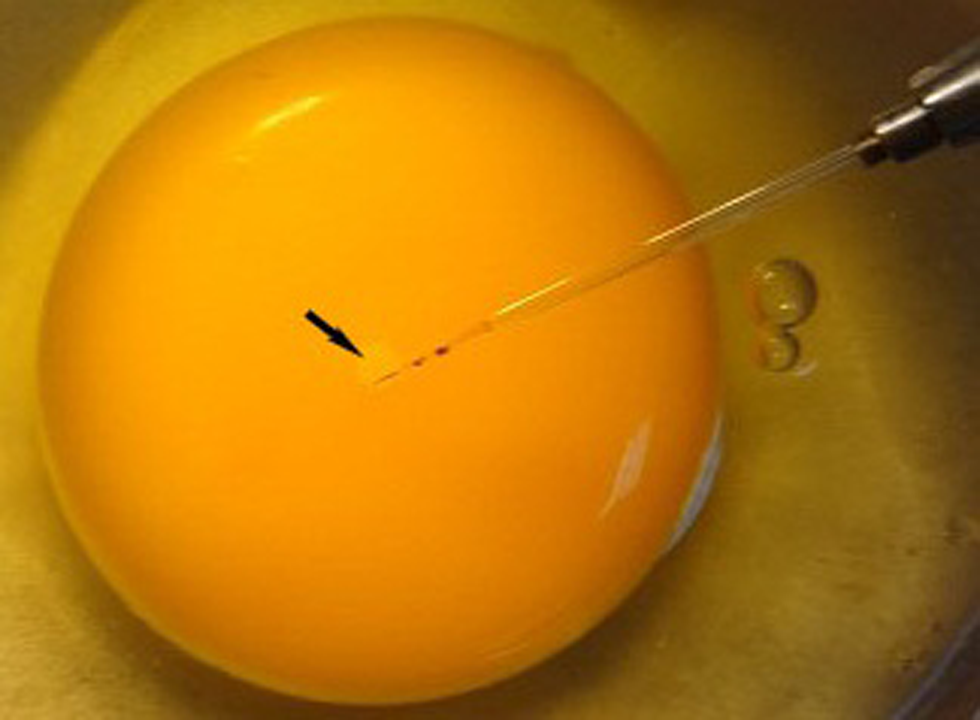

Supplement: Figure S3 — A photo showing the microinjection to subgerminal cavity of a freshly laid quail egg at Stage X. The arrow indicates the subgerminal cavity. (TIF) [file pone.0050817.s003.tif]

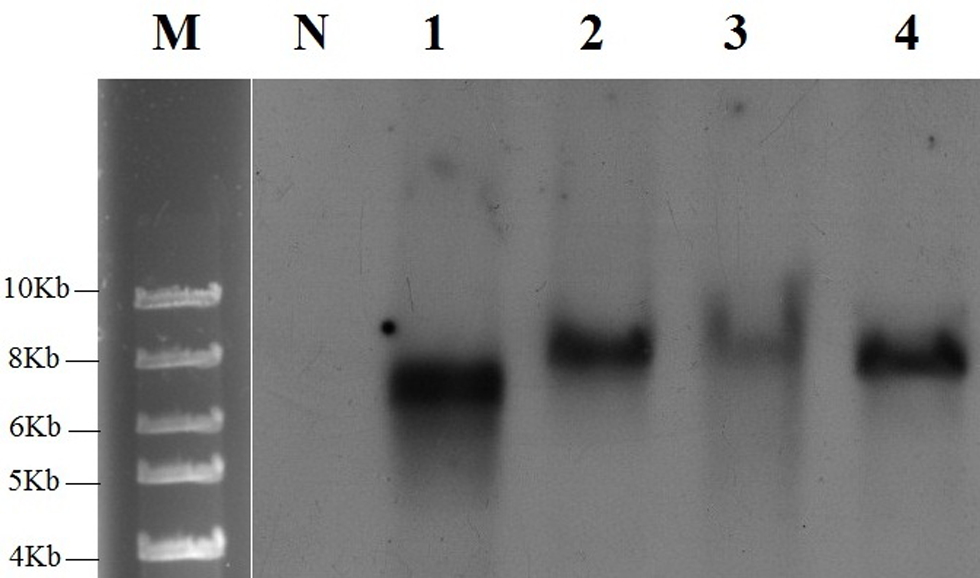

Supplement: Figure S4 — Southern blot of the 4 positive G1 transgenic quails from Subgerminal cavity injection group. The genomice DNA extracted from blood was digested with EcoRI. Probe used in this experiment was a 876 bp DIG-labeled probe asdescribe in materials and methods. N, nontransgenic quail; Lanes 1–4 indicate G1 transgenic quails 1–4. (TIF) [file pone.0050817.s004.tif]
