# Supplementary material for: Transgenic Quail Production by Microinjection of Lentiviral Vector into the Early Embryo Blood Vessels
Source: PLoS One. 2012 Dec 12;7(12):e50817. doi: 10.1371/journal.pone.0050817 (PMC3520935; doi:10.1371/journal.pone.0050817)
Supplement: Text S1 — The exact sequences of lentiviral vector pGCL-eGFP. (DOC) [file pone.0050817.s005.doc]

Text S1 The exact sequences of lentiviral vector pGCL-eGFP

1 gcttaagcggtcgacggatcgggagatctcccgatcccctatggtgcactctcagtacaa

61 tctgctctgatgccgcatagttaagccagtatctgctccctgcttgtgtgttggaggtcg

121 ctgagtagtgcgcgagcaaaatttaagctacaacaaggcaaggcttgaccgacaattgca

181 tgaagaatctgcttagggttaggcgttttgcgctgcttcgcgatgtacgggccagatata

241 cgcgttgacattgattattgactagttattaatagtaatcaattacggggtcattagttc

301 atagcccatatatggagttccgcgttacataacttacggtaaatggcccgcctggctgac

361 cgcccaacgacccccgcccattgacgtcaataatgacgtatgttcccatagtaacgccaa

421 tagggactttccattgacgtcaatgggtggagtatttacggtaaactgcccacttggcag

481 tacatcaagtgtatcatatgccaagtacgccccctattgacgtcaatgacggtaaatggc

541 ccgcctggcattatgcccagtacatgaccttatgggactttcctacttggcagtacatct

601 acgtattagtcatcgctattaccatggtgatgcggttttggcagtacatcaatgggcgtg

661 gatagcggtttgactcacggggatttccaagtctccaccccattgacgtcaatgggagtt

721 tgttttggcaccaaaatcaacgggactttccaaaatgtcgtaacaactccgccccattga

781 cgcaaatgggcggtaggcgtgtacggtgggaggtctatataagcagcgcgttttgcctgt

841 actgggtctctctggttagaccagatctgagcctgggagctctctggctaactagggaac

901 ccactgcttaagcctcaataaagcttgccttgagtgcttcaagtagtgtgtgcccgtctg

961 ttgtgtgactctggtaactagagatccctcagacccttttagtcagtgtggaaaatctct

1021 agcagtggcgcccgaacagggacttgaaagcgaaagggaaaccagaggagctctctcgac

1081 gcaggactcggcttgctgaagcgcgcacggcaagaggcgaggggcggcgactggtgagta

1141 cgccaaaaattttgactagcggaggctagaaggagagagatgggtgcgagagcgtcagta

1201 ttaagcgggggagaattagatcgcgatgggaaaaaattcggttaaggccagggggaaaga

1261 aaaaatataaattaaaacatatagtatgggcaagcagggagctagaacgattcgcagtta

1321 atcctggcctgttagaaacatcagaaggctgtagacaaatactgggacagctacaaccat

1381 cccttcagacaggatcagaagaacttagatcattatataatacagtagcaaccctctatt

1441 gtgtgcatcaaaggatagagataaaagacaccaaggaagctttagacaagatagaggaag

1501 agcaaaacaaaagtaagaccaccgcacagcaagcggccggccgcgctgatcttcagacct

1561 ggaggaggagatatgagggacaattggagaagtgaattatataaatataaagtagtaaaa

1621 attgaaccattaggagtagcacccaccaaggcaaagagaagagtggtgcagagagaaaaa

1681 agagcagtgggaataggagctttgttccttgggttcttgggagcagcaggaagcactatg

1741 ggcgcagcgtcaatgacgctgacggtacaggccagacaattattgtctggtatagtgcag

1801 cagcagaacaatttgctgagggctattgaggcgcaacagcatctgttgcaactcacagtc

1861 tggggcatcaagcagctccaggcaagaatcctggctgtggaaagatacctaaaggatcaa

1921 cagctcctggggatttggggttgctctggaaaactcatttgcaccactgctgtgccttgg

1981 aatgctagttggagtaataaatctctggaacagatttggaatcacacgacctggatggag

2041 tgggacagagaaattaacaattacacaagcttaatacactccttaattgaagaatcgcaa

2101 aaccagcaagaaaagaatgaacaagaattattggaattagataaatgggcaagtttgtgg

2161 aattggtttaacataacaaattggctgtggtatataaaattattcataatgatagtagga

2221 ggcttggtaggtttaagaatagtttttgctgtactttctatagtgaatagagttaggcag

2281 ggatattcaccattatcgtttcagacccacctcccaaccccgaggggacccgacaggccc

2341 gaaggaatagaagaagaaggtggagagagagacagagacagatccattcgattagtgaac

2401 ggatcggcactgcgtgcgccaattctgcagacaaatggcagtattcatccacaattttaa

2461 aagaaaaggggggattggggggtacagtgcaggggaaagaatagtagacataatagcaac

2521 agacatacaaactaaagaattacaaaaacaaattacaaaaattcaaaattttcgggttta

2581 ttacagggacagcagagatccagtttggttagtaccgggcccgctctagactcgagcggc

2641 cgcccccttcaccgagggcctatttcccatgattccttcatatttgcatatacgatacaa

2701 ggctgttagagagataattggaattaatttgactgtaaacacaaagatattagtacaaaa

2761 tacgtgacgtagaaagtaataatttcttgggtagtttgcagttttaaaattatgttttaa

2821 aatggactatcatatgcttaccgtaacttgaaagtatttcgatttcttggctttatatat

2881 cttgtggaaaggacgaaacaccggtccgcaggtatgcacgcgtgaattcggatccattag

2941 gcggccgcgtggataaccgtattaccgccatgcattagttattaatagtaatcaattacg

3001 gggtcattagttcatagcccatatatggagttccgcgttacataacttacggtaaatggc

3061 ccgcctggctgaccgcccaacgacccccgcccattgacgtcaataatgacgtatgttccc

3121 atagtaacgccaatagggactttccattgacgtcaatgggtggagtatttacggtaaact

3181 gcccacttggcagtacatcaagtgtatcatatgccaagtacgccccctattgacgtcaat

3241 gacggtaaatggcccgcctggcattatgcccagtacatgaccttatgggactttcctact

3301 tggcagtacatctacgtattagtcatcgctattaccatggtgatgcggttttggcagtac

3361 atcaatgggcgtggatagcggtttgactcacggggatttccaagtctccaccccattgac

3421 gtcaatgggagtttgttttggcaccaaaatcaacgggactttccaaaatgtcgtaacaac

3481 tccgccccattgacgcaaatgggcggtaggcgtgtacggtgggaggtctatataagcaga

3541 gctggtttagtgaaccgtcagatccgctagcgctaccggacgccaccatggtgagcaagg

3601 gcgaggagctgttcaccggggtggtgcccatcctggtcgagctggacggcgacgtaaacg

3661 gccacaagttcagcgtgtccggcgagggcgagggcgatgccacctacggcaagctgaccc

3721 tgaagttcatctgcaccaccggcaagctgcccgtgccctggcccaccctcgtgaccaccc

3781 tgacctacggcgtgcagtgcttcagccgctaccccgaccacatgaagcagcacgacttct

3841 tcaagtccgccatgcccgaaggctacgtccaggagcgcaccatcttcttcaaggacgacg

3901 gcaactacaagacccgcgccgaggtgaagttcgagggcgacaccctggtgaaccgcatcg

3961 agctgaagggcatcgacttcaaggaggacggcaacatcctggggcacaagctggagtaca

4021 actacaacagccacaacgtctatatcatggccgacaagcagaagaacggcatcaaggtga

4081 acttcaagatccgccacaacatcgaggacggcagcgtgcagctcgccgaccactaccagc

4141 agaacacccccatcggcgacggccccgtgctgctgcccgacaaccactacctgagcaccc

4201 agtccgccctgagcaaagaccccaacgagaagcgcgatcacatggtcctgctggagttcg

4261 tgaccgccgccgggatcactctcggcatggacgagctgtacaagtaacgtcgagggacct

4321 aataacttcgtatagcatacattatacgaagttatacatgtttaagggttccggttccac

4381 taggtacaattcgatatcaagcttatcgataatcaacctctggattacaaaatttgtgaa

4441 agattgactggtattcttaactatgttgctccttttacgctatgtggatacgctgcttta

4501 atgcctttgtatcatgctattgcttcccgtatggctttcattttctcctccttgtataaa

4561 tcctggttgctgtctctttatgaggagttgtggcccgttgtcaggcaacgtggcgtggtg

4621 tgcactgtgtttgctgacgcaacccccactggttggggcattgccaccacctgtcagctc

4681 ctttccgggactttcgctttccccctccctattgccacggcggaactcatcgccgcctgc

4741 cttgcccgctgctggacaggggctcggctgttgggcactgacaattccgtggtgttgtcg

4801 gggaaatcatcgtcctttccttggctgctcgcctgtgttgccacctggattctgcgcggg

4861 acgtccttctgctacgtcccttcggccctcaatccagcggaccttccttcccgcggcctg

4921 ctgccggctctgcggcctcttccgcgtcttcgccttcgccctcagacgagtcggatctcc

4981 ctttgggccgcctccccgcatcgataccgtcgacctcgatcgagacctagaaaaacatgg

5041 agcaatcacaagtagcaatacagcagctaccaatgctgattgtgcctggctagaagcaca

5101 agaggaggaggaggtgggttttccagtcacacctcaggtacctttaagaccaatgactta

5161 caaggcagctgtagatcttagccactttttaaaagaaaaggggggactggaagggctaat

5221 tcactcccaacgaagacaagatatccttgatctgtggatctaccacacacaaggctactt

5281 ccctgattggcagaactacacaccagggccagggatcagatatccactgacctttggatg

5341 gtgctacaagctagtaccagttgagcaagagaaggtagaagaagccaatgaaggagagaa

5401 cacccgcttgttacaccctgtgagcctgcatgggatggatgacccggagagagaagtatt

5461 agagtggaggtttgacagccgcctagcatttcatcacatggcccgagagctgcatccgga

5521 ctgtactgggtctctctggttagaccagatctgagcctgggagctctctggctaactagg

5581 gaacccactgcttaagcctcaataaagcttgccttgagtgcttcaagtagtgtgtgcccg

5641 tctgttgtgtgactctggtaactagagatccctcagacccttttagtcagtgtggaaaat

5701 ctctagcagcatgtgagcaaaaggccagcaaaaggccaggaaccgtaaaaaggccgcgtt

5761 gctggcgtttttccataggctccgcccccctgacgagcatcacaaaaatcgacgctcaag

5821 tcagaggtggcgaaacccgacaggactataaagataccaggcgtttccccctggaagctc

5881 cctcgtgcgctctcctgttccgaccctgccgcttaccggatacctgtccgcctttctccc

5941 ttcgggaagcgtggcgctttctcatagctcacgctgtaggtatctcagttcggtgtaggt

6001 cgttcgctccaagctgggctgtgtgcacgaaccccccgttcagcccgaccgctgcgcctt

6061 atccggtaactatcgtcttgagtccaacccggtaagacacgacttatcgccactggcagc

6121 agccactggtaacaggattagcagagcgaggtatgtaggcggtgctacagagttcttgaa

6181 gtggtggcctaactacggctacactagaagaacagtatttggtatctgcgctctgctgaa

6241 gccagttaccttcggaaaaagagttggtagctcttgatccggcaaacaaaccaccgctgg

6301 tagcggtggtttttttgtttgcaagcagcagattacgcgcagaaaaaaaggatctcaaga

6361 agatcctttgatcttttctacggggtctgacgctcagtggaacgaaaactcacgttaagg

6421 gattttggtcatgagattatcaaaaaggatcttcacctagatccttttaaattaaaaatg

6481 aagttttaaatcaatctaaagtatatatgagtaaacttggtctgacagttaccaatgctt

6541 aatcagtgaggcacctatctcagcgatctgtctatttcgttcatccatagttgcctgact

6601 ccccgtcgtgtagataactacgatacgggagggcttaccatctggccccagtgctgcaat

6661 gataccgcgagacccacgctcaccggctccagatttatcagcaataaaccagccagccgg

6721 aagggccgagcgcagaagtggtcctgcaactttatccgcctccatccagtctattaattg

6781 ttgccgggaagctagagtaagtagttcgccagttaatagtttgcgcaacgttgttgccat

6841 tgctacaggcatcgtggtgtcacgctcgtcgtttggtatggcttcattcagctccggttc

6901 ccaacgatcaaggcgagttacatgatcccccatgttgtgcaaaaaagcggttagctcctt

6961 cggtcctccgatcgttgtcagaagtaagttggccgcagtgttatcactcatggttatggc

7021 agcactgcataattctcttactgtcatgccatccgtaagatgcttttctgtgactggtga

7081 gtactcaaccaagtcattctgagaatagtgtatgcggcgaccgagttgctcttgcccggc

7141 gtcaatacgggataataccgcgccacatagcagaactttaaaagtgctcatcattggaaa

7201 acgttcttcggggcgaaaactctcaaggatcttaccgctgttgagatccagttcgatgta

7261 acccactcgtgcacccaactgatcttcagcatcttttactttcaccagcgtttctgggtg

7321 agcaaaaacaggaaggcaaaatgccgcaaaaaagggaataagggcgacacggaaatgttg

7381 aatactcatactcttcctttttcaatattattgaagcatttatcagggttattgtctcat

7441 gagcggatacatatttgaatgtatttagaaaaataaacaaataggggttccgcgcacatt

7501 tccccgaaaagtgccacctg ac
